# Magnetization transfer and diffusion tensor imaging in dogs with intervertebral disk herniation

**DOI:** 10.1111/jvim.15899

**Published:** 2020-10-02

**Authors:** Richard L. Shinn, Theresa E. Pancotto, Krystina L. Stadler, Stephen R. Werre, John H. Rossmeisl

**Affiliations:** ^1^ Department of Small Animal Clinical Sciences Virginia‐Maryland College of Veterinary Medicine, Virginia Polytechnic Institute and State University Blacksburg Virginia USA; ^2^ Summit Veterinary Referral Center Tacoma Washington USA; ^3^ Laboratory for Study Design and Statistical Analysis, Virginia‐Maryland Regional College of Veterinary Medicine, Virginia Polytechnic Institute and State University Blacksburg Virginia USA

**Keywords:** axial diffusivity, fractional anisotropy, intervertebral disk disease, magnetic resonance imaging, magnetization transfer ratio, mean diffusivity, radial diffusivity, spinal cord injury

## Abstract

**Background:**

Quantitative magnetic resonance imaging (QMRI) techniques of magnetization transfer ratio (MTR) and diffusion tensor imaging (DTI) provide microstructural information about the spinal cord.

**Objective:**

Compare neurologic grades using the modified Frankel scale with MTR and DTI measurements in dogs with thoracolumbar intervertebral disk herniation (IVDH).

**Animals:**

Fifty‐one dogs with thoracolumbar IVDH.

**Methods:**

Prospective cohort study. Quantitative MRI measurements of the spinal cord were obtained at the region of compression. A linear regression generalized estimating equations model was used to compare QMRI measurements between different neurological grades after adjusting for age, weight, duration of clinical signs, and lesion location.

**Results:**

Grade 5 (.79  ×  10^−3^ mm^2^/s [median], .43−.91 [range]) and axial (1.47 × 10^−3^ mm^2^/s, .58−1.8) diffusivity were lower compared to grades 2 (1.003, .68−1.36; *P* = .02 and 1.81 × 10^−3^ mm^2^/s, 1.36−2.12; *P* < .001, respectively) and 3 (1.07 × 10^−3^ mm^2^/s, .77−1.5; *P* = .04 and 1.92 × 10^−3^ mm^2^/s, 1.83−2.37;*P* < .001, respectively). Compared to dogs with acute myelopathy, chronic myelopathy was associated with higher mean (1.02 × 10^−3^ mm^2^/s, .77−1.36 vs. .83 × 10^−3^ mm^2^/s, .64−1.5; *P* = .03) and radial diffusivity (.75 × 10^−3^ mm^2^/s, .38−1.04 vs. .44 × 10^−3^ mm^2^/s, .22−1.01; *P* = .008) and lower MTR (46.76, 31.8−56.43 vs. 54.4, 45.2−62.27; *P* = .004) and fractional anisotropy (.58, .4−0.75 vs. .7, .46−.85; *P* = .02). Fractional anisotropy was lower in dogs with a T2‐weighted intramedullary hyperintensity compared to those without (.7, .45−.85 vs. .54, .4−.8; *P* = .01).

**Conclusion and Clinical Relevance:**

Mean diffusivity and AD could serve as surrogates of severity of spinal cord injury and are complementary to the clinical exam in dogs with thoracolumbar IVDH.

**Abbreviations**
AD: axial diffusivity
CNS: central nervous system
DICOM: Digital Imaging and Communications in Medicine
DTI: diffusion tensor imaging
FA: fractional anisotropy
IVDH: intervertebral disk herniation
MD: mean diffusivity
MRI: magnetic resonance imaging
MT: magnetization transfer
MTR: magnetization transfer ratio
QMRI: quantitative magnetic resonance imaging
RD: radial diffusivity
ROI: region of interest
SCI: spinal cord injury
SCT: spinal cord toolbox
T2W‐IH: T2‐weighted intramedullary hyperintensity

## 1 INTRODUCTION

Intervertebral disk herniation (IVDH) is considered to be the most common cause of spinal cord injury (SCI) in dogs and prognosis directly correlates to neurologic grade.^1^ After severe SCI and loss of deep pain perception neuronal death can ensue; alternatively injured neurons initially might not generate action potentials but will subsequently recover leading to prognostic uncertainty of dogs with loss of deep pain perception.^2^, ^3^ Magnetic resonance imaging (MRI) is the ideal tool to noninvasively evaluate the central nervous system (CNS).^4^ Despite a high sensitivity in detection of CNS abnormalities, conventional MRI provides limited assessment of SCI severity in dogs with IVDH.^5^, ^6^ In dogs with thoracolumbar IVDH, T2‐weighted intramedullary hyperintensity (T2W‐IH) correlates negatively with prognosis.^5^, ^6^ Morphometric measurements, such as the degree of spinal cord compression, vertebral canal compression ratio, and compressive length of the spinal cord, are not predictive of outcome.^5^, ^7^, ^8^

Quantitative MRI (QMRI) techniques of magnetization transfer (MT) and diffusion tensor imaging (DTI) have the ability to noninvasively assess myelination and axonal integrity, and are highly predictive of histopathological changes.^9^, ^10^ Calculation of the magnetization transfer ratio (MTR) provides a measure of the rate of magnetization exchange between the hydrogen protons in low protein fluid and those protons bound to macromolecules in tissue.^11^, ^12^ Magnetization transfer alterations are thought to be directly related to myelin damage,^9^ and have been evaluated in people with myelopathy.^13^Quantitative DTI measurements thought to be of clinical importance in SCI include fractional anisotropy (FA), mean diffusivity (MD), axial diffusivity (AD), and radial diffusivity (RD). Variations of FA and MD represent demyelination and changes to axonal integrity.^14^ Axial diffusivity is thought to be a surrogate of axonal integrity; RD a surrogate of myelin disintegration.^14^ These quantitative DTI measurements have been evaluated in dogs with experimentally induced SCI, but only FA and MD have been evaluated in dogs with IVDH.^15^

The goal of our study was to evaluate MTR and DTI in dogs with thoracolumbar IVDH. We hypothesized that MTR and FA would be higher, while MD, AD, and RD would be lower in dogs with more severe neurologic dysfunction. Within the CNS, MTR increase with acute CNS injury, then decrease with chronic CNS injury.^16^ We therefore hypothesized that MTR would be higher in dogs with acute and subacute SCI compared to dogs with chronic SCI. Alteration in FA have been associated with the presence of a T2W‐IH^17^ and we hypothesized that FA would be higher in dogs with a T2W‐IH compared to dogs without a T2W‐IH.

## 2 MATERIALS AND METHODS

This was a prospective cohort study. Inclusion criteria included dogs of any age, sex, or breed that were presented to the Virginia Maryland College of Veterinary Medicine between May 2017 and December 2018. Dog had to have a thoracolumbar myelopathy diagnosed with IVDH on MRI, and had an IVDH confirmed with surgery. Hospital board approval and owner informed consent were obtained prior to enrollment.

Dogs were grouped based on the severity of their neurological grade at presentation using a modified Frankel scale (Table 1).^18^, ^19^, ^20^ To evaluate deep pain perception, the distal limb or nail bed was cross‐clamped with hemostats (either using the nose or the handle of the hemostats) and then observing a conscious reaction to the painful stimulus or an increase in heart rate, respiratory rate, or pupil size.^21^

**TABLE 1:** Modified Frankel score for thoracolumbar intervertebral disk herniation

Neurologic grade	Neurologic signs
0	Normal examination
1	Normal ambulation with spinal hyperpathia
2	Ambulatory paraparesis with proprioceptive ataxia
3	Nonambulatory paraparesis
4	Paraplegia with intact deep pain perception
5	Paraplegia without deep pain perception

All dogs in the study were anesthetized for MRI. Anesthesia protocol was at the discretion of the anesthesiologist. Magnetic resonance imaging was performed using a 1.5T magnet (Philips Intera 1.5T MRI scanner, Cleveland, Ohio). An 8‐channel knee coil or single channel spine coil was used for image acquisition. Dogs underwent conventional spine MRI, including at least T2 weighted transverse and sagittal, and dorsal Short Tau Inversion Reverory (STIR) sequences. Additional sequences such as T1 weighted and T2* weighted images and contrast media administration were dependent on clinician and lesion. Magnetization transfer ratio and DTI sequences were performed after conventional precontrast sequences (Table 2). Surgery was then performed to confirm diagnosis of IVDH.

**TABLE 2:** MRI acquisition protocol

Imaging type	Technical details
T2WI	Avg TR/TE = 3000/120, matrix = 200 to 388 × 190 to 348, FOV = 140 to 288 × 140 to 288 mm^2^, flip angle = 90°, acq voxel MPS = 0.7 × 0.74 × 3.0 mm, slice gap = 0.3 mm, sagittal orientation
STIR	Avg TR/TE = 2265/30, matrix = 168 to 252 × 147 to 215, FOV = 150 to 250 × 156 to 275 mm^2^, acq voxel MPS = 0.89 × 1.12 × 3.0 mm, slice gap = 0.3 mm, dorsal orientation
DTI	Modified Phillips DTI protocol (DTI_medium_isosense), avg TR/TE = 4917/72, matrix = 64 × 133, gradient directions = 15 nonparallel diffusion, *b* = 800 s/mm^2^, acq voxel MPS = 2.03 × 2.02 × 2.00 mm, transverse orientation. Average DTI scan time was 10 min.
3D MTR	FOV = 130 × 264 mm^2^, matrix = 144 × 293, TR/TE = 37/4.7, flip angle = 8°, acq voxel MPS = 0.9 × 0.9 × 4.0 mm, magnetization transfer contrast = off resonance, transverse orientation. Average 3D MTR scan time was 7 min.

Abbreviations: DTI, diffusion tensor imaging; FOV, field of view; MRI, magnetic resonance imaging; MTR, magnitization transfer ratio; MPS, voxel sizes in measurement; STIR, short tau inversion recovery; T2WI, T2 weighted image; TE, echo time; TR, repetition time.

**FIGURE 1:**
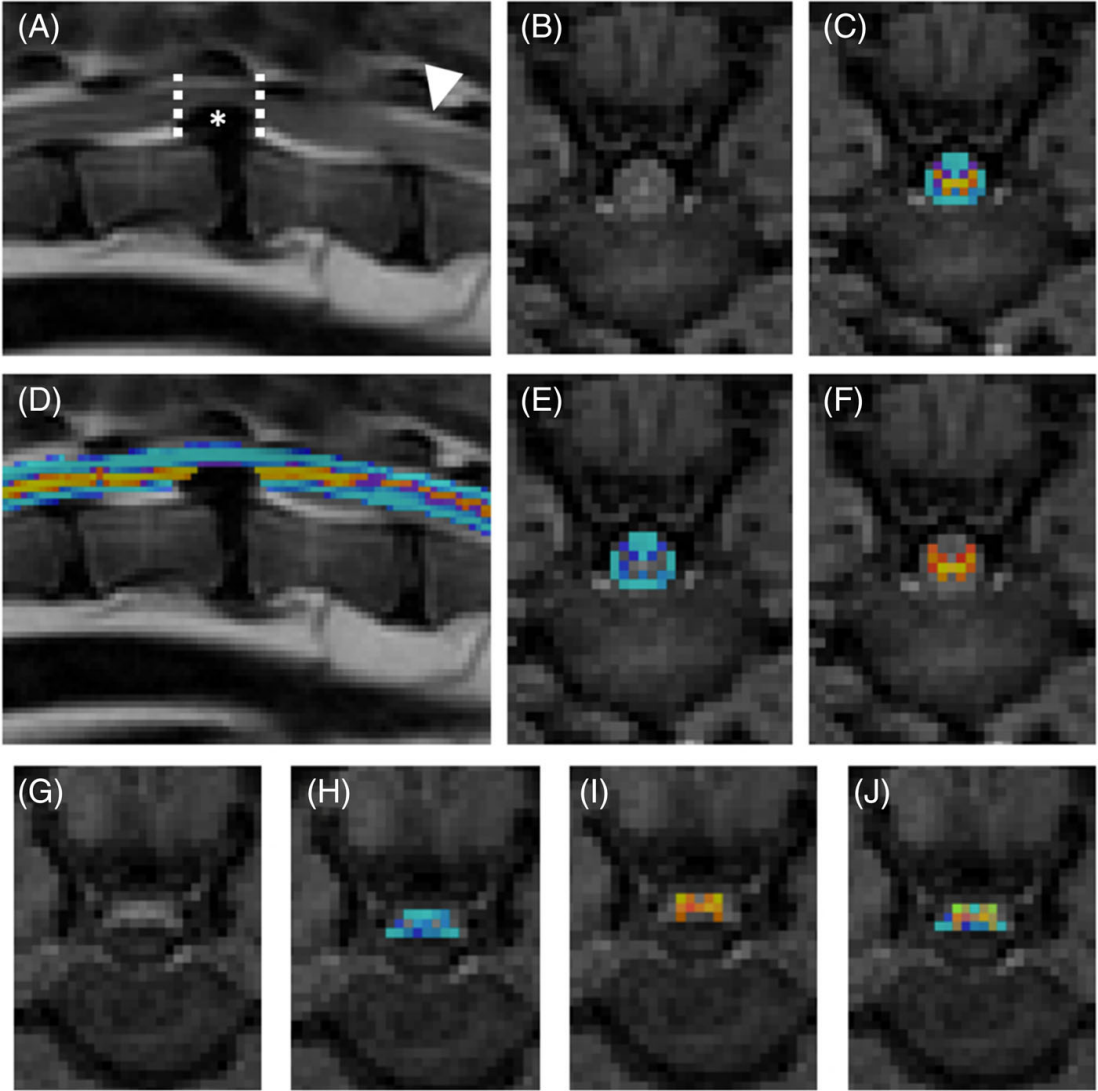
Example of MRI T2 (A, D) and MTR (B, C, E, F‐J) segmentation with Spinal Cord Toolbox. A, T2‐weighted sagittal of L1‐L2 intervertebral disk herniation. The asterisk indicates the disk herniation. The dashed lines represent the cranial and caudal boarders for the spinal cord compression and where MTR was obtained (images G‐J). The arrowhead indicates a normal region of the spinal cord for comparison (images B, C, E, and F). D, WM (blue) and GM (yellow and orange) segmentation from SCT software overlaid on the T2‐weighted sagittal sequence. B, Transverse MT image caudal to the region of compression. C, WM and GM segmentation combined overlaid on the MT transverse image representing the region of interest calculated by SCT. E, WM segmentation overlaid on the MT transverse image. F, GM segmentation overlaid on the MT transverse image. G, MT transverse image at the level of spinal cord compression. H, WM segmentation overlaid on the MT transverse image. I, GM segmentation overlaid on the MT transverse image. J, WM and GM segmentation combined overlaid on the MT transverse image representing the region of interest for MTR measurement calculated by SCT. GM, gray matter; MRI, magnetic resonance imaging; MT, magnetization transfer image with saturation pulse; MTR, magnetization transfer ratio; SCT, Spinal Cord Toolbox; WM, white matter

**FIGURE 2:**
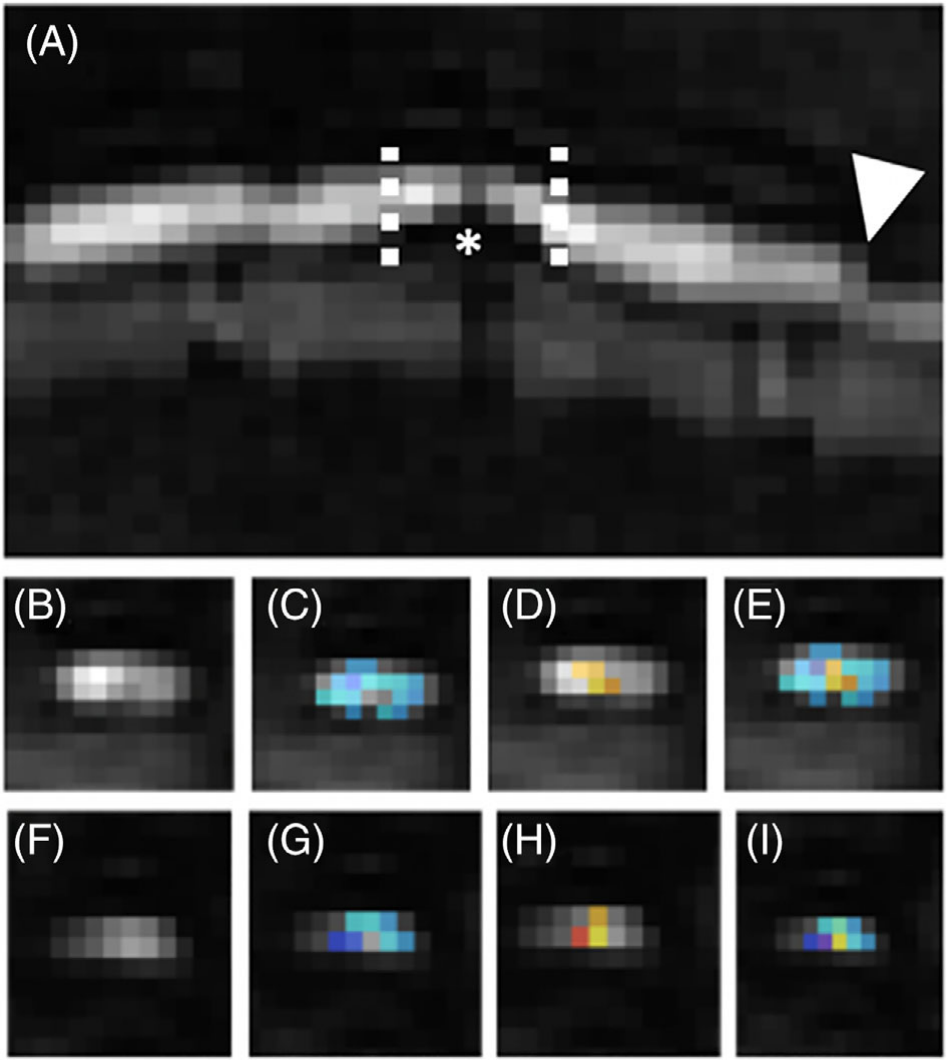
Example of DTI segmentation with Spinal Cord Toolbox in the same dog as Figure 1. A, Sagittal DTI of L1‐L2 intervertebral disk herniation. The asterisk indicates the disk herniation. The dashed lines represent the cranial and caudal boarders for the spinal cord compression and where DTI measurements were obtained (images F‐I). The arrowhead indicates a normal region of the spinal cord for (images B‐E). B, Transverse DTI caudal to the region of compression. C, WM segmentation from SCT software overlaid transverse DTI. D, GM segmentation overlaid transverse DTI. E, WM (blue) and GM (orange) segmentation combined overlaid transverse DTI representing the region of interest for FA, MD, AD, and RD calculated by SCT. F, Transverse DTI at the area of spinal cord compression. G, WM segmentation overlaid transverse DTI. H, GM segmentation overlaid transverse DTI. I, WM (blue) and GM (orange) segmentation combined overlaid transverse DTI representing the region of interest for FA, MD, AD, and RD calculated by SCT. AD, axial diffusivity; DTI, diffusion tensor imaging; FA, fractional anisotropy; GM, gray matter; MD, mean diffusivity; RD, radial diffusivity; SCT, Spinal Cord Toolbox; WM, white matter

The images were transferred using Digital Imaging and Communications in Medicine (DICOM) format to a secondary workstation using open‐source software (Horos‐64bit version 3.3.6) for image viewing, and Spinal Cord Toolbox (SCT) version 3.1.1 for QMRI processing (NeuroPoly Lab, Institute of Biomedical Engineering, Polytechnique Montreal, Montreal, Canada).^22^ The region of compression and presence of a T2W‐IH were determined by T2W sagittal and transverse images, similar to a previous study.^23^ An average MTR, FA, MD, AD, and RD were obtained over the epicenter of compression (Figures 1 and 2). If a T2W‐IH was present, this did not change where QMRI measurements were performed. For SCT, DICOM images were converted to nii files using dcm2nii (64bit BSD) open source software. Once converted, nii images were processed using SCT to obtain the QMRI measurements of MTR, FA, MD, AD, and RD (Figures 1 and 2). Spinal Cord Toolbox obtains QMRI measurements similar to utilizing region of interest (ROI) but provides a more accurate estimation of the QMRI measurement as it is able to account for partial volume effect using Gaussian mixture modeling.^22^ Following processing, images were inspected using the open source software FSLView (version 3.2.0) to confirm SCT segmentation (Figures 1 and 2). All images were reviewed by the primary author. In FSLView, the images appear as larger pixels.

In addition to the modified Frankel grade, the sex, age, body weight, longitudinal location of the IVDH in the vertebral column (ie, affected spinal cord segment), duration of clinical signs, and presence or absence T2W‐IH was recorded from the medical record of each case such that these variables could be examined for associations with QMRI measurements. Duration of clinical signs was divided into 3 categories, acute SCI (0‐3 days), subacute SCI (3 days‐3 weeks), and chronic SCI (>3 weeks).^24^ Longitudinal IVDH location was included as DTI measurements have been shown to differ between spinal cord segments.^25^

### 2.1 Statistical analysis

Outcomes were the QMRI measurements MTR, FA, MD, AD, and RD. Primary exposure of interest was neurological grade. Baseline variables included sex, age, body weight, duration of clinical signs (acute, subacute, chronic), lesion location (between the T3 and S3 spinal cord segments), and presence or absence of T2W‐IH. Normal probability plots showed that QMRI measurements, age, and body weight were skewed. Accordingly, continuous data were summarized as median (minimum, maximum) while categorical data were summarized as counts. Bivariable associations between QMRI measurements (1 measurement at a time) and baseline variables were assessed using the Kruskal‐Wallis test (sex, duration of clinical signs, and lesion location), Spearman's correlation coefficient (age and body weight), and the Wilcoxon rank sum test (T2W‐IH). Bivariable associations between QMRI measurements (1 measurement at a time) and neurological grade were assessed using the Krukal‐Wallis test followed by Dunn's procedure for multiple comparisons. To evaluate effects of neurological score after adjusting for baseline variables (significant on bivariable analysis), QMRI measurements were further analyzed using multivariable linear generalized estimating equations (GEEs). Two‐way comparisons between neurological scores within the GEE model were adjusted for multiple comparisons using Tukey's procedure. Statistical significance was set to *P* < .05. All analyses were performed using SAS version 9.4 (Cary, North Carolina).

## 3 RESULTS

A total of 52 MRIs were performed on 51 dogs included in the study. Breeds included miniature dachshund (n = 25), mixed breed (n = 4), beagle (n = 3), coonhound (n = 2), German shepherd (n = 2), and 1 for each of the following breeds: basset hound, Boston terrier, English bulldog, chihuahua, cocker spaniel, corgi, Doberman, Lhasa Apso, miniature schnauzer, red bone hound, Sealyham terrier, Shih Tzu, Staffordshire terrier, toy poodle, and Yorkshire terrier. Twenty‐three dogs were spayed females, 18 dogs were neutered males, 8 dogs were intact males, and 2 dogs were intact females. The age range was 3 to 14 years (median of 6 years), and the body weight range was 3.6 to 42 kg (median 8.25 kg). The distribution of presenting modified Frankel grades were: grade 2 (n = 14), grade 3 (n = 13), grade 4 (n = 10), and grade 5 (n = 15). Twenty‐nine dogs presented with acute SCI, 14 with subacute SCI, and 8 with chronic SCI.

When images were evaluated, the mean pixel value for MTR was 23 and for DTI was 10 (Figures 1 and 2). Bivariable analyses did not identify significant differences between sex and MTR, FA, MD, AD, or RD. Significant correlations were found with age and MD (*r* = 0.67, *P* < .001), AD (*r* = 0.41, *P* = .002), RD (*r* = 0.50, *P* < .001), and FA (*r* = −0.38, *P* = .006). A significant correlation of age with MTR (*r* = 0.16, *P* = .26) was not found. Significant correlations with body weight were found with MD (*r* = 0.40, *P* = .003), AD (*r* = 0.31, *P* = .03), RD (*r* = 0.30, *P* = .03), but not MTR (*r* = 0.2, *P* = .15) and FA (*r* = −0.13, *P* = .34). Longitudinal location of IVDH was significantly associated with MTR (*P* = .01) and MD (*P* = .008), but not AD (*P* = .29), RD (*P* = .28), or FA (*P* = .25).

**FIGURE 3:**
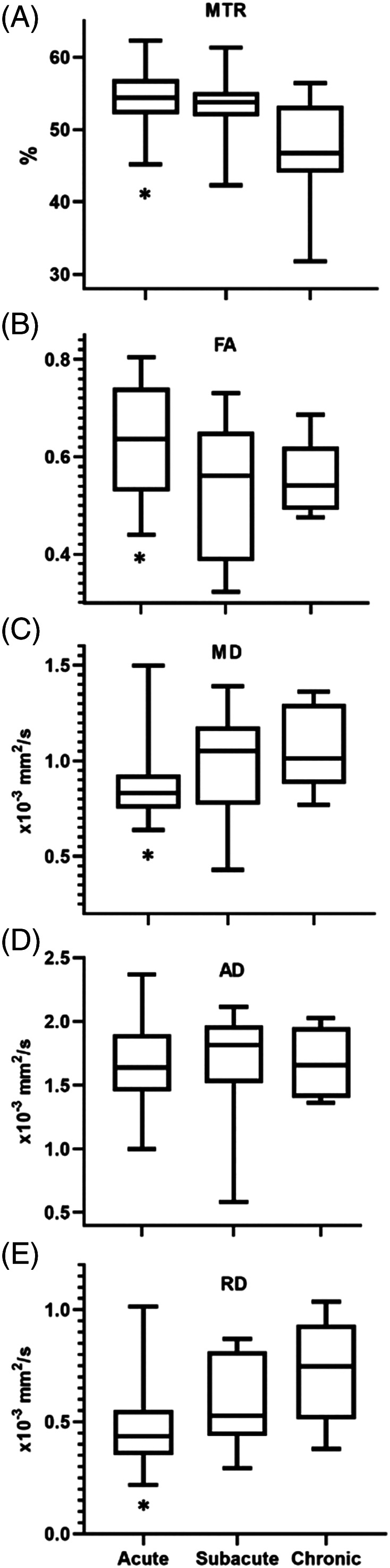
Association of SCI duration with MTR (A), FA (B), MD (C), AD (D), and RD (E). Twenty‐nine dogs presented with acute SCI, 14 with subacute SCI, and 8 with chronic SCI. An asterisk indicates statistical significance of *P* < .05 when compared to chronic. AD, axial diffusivity; FA, fractional anisotropy; MD, mean diffusivity; MTR, magnetization transfer ratio; RD, radial diffusivity; SCI, spinal cord injury

Magnetization transfer ratio (*P* = .004) and FA (*P* = .02) were significantly lower in dogs with chronic SCI compared to dogs with acute SCI, but not when compared to subacute SCI (Figure 3). Mean diffusivity (*P* = .03) and RD (*P* = .008) were significantly higher in dogs with chronic SCI compared to acute SCI, but not when compared to subacute SCI (Figure 3). Axial diffusivity was not significantly different when chronic SCI was compared to acute or subacute SCI. FA was significantly lower in dogs where a T2W‐IH was present (*P* = .01; Table 3) compared to dogs without a T2W‐IH. Magnetization transfer ratio, MD, AD, and RD were not significantly different in dogs with or without T2W‐IH.

**TABLE 3:** QMRI measurements with and without the presence of a T2W‐IH

	T2W‐IH present (n = 11)	T2W‐IH absent (n = 42)	*P* value
MTR	55.34	53.08	.34
	(45.48‐61.16)	(31.79‐62.27)	
FA	0.54	0.70	.01*
	(0.40‐0.80)	(0.45‐0.85)	
MD	0.91	0.88	.59
	(0.43‐1.49)	(0.64‐1.36)	
AD	1.59	1.75	.7
	(0.58‐2.40)	(1.04‐2.16)	
RD	0.74	0.46	.25
	(0.28‐1.04)	(0.22‐1.01)	

*Note*: Data are expressed as median (range). MD, AD, and RD units are ×10^−3^ mm^2^/s. * indicates statistical significance of *P* < .05.

Abbreviations: AD, axial diffusivity; FA, fractional anisotropy; MD, mean diffusivity; MTR, magnetization transfer ratio; QMRI, quantitative magnetic resonance imaging; RD, radial diffusivity; T2W‐IH, T2‐weighted intramedullary hyperintensity.

**FIGURE 4:**
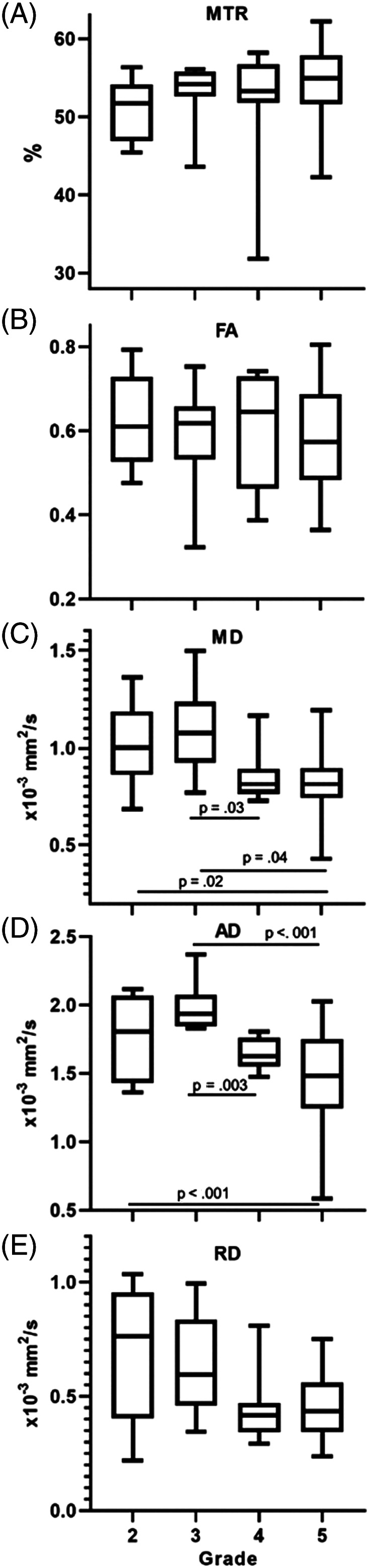
A, MTR, B, FA, C, MD, D, AD, and, E, RD compared between different neurologic grades. For grade 2 n = 14, grade 3 n = 13, grade 4 n = 10, and grade 5 n = 15. MD and AD were significantly different between grades on multivariable analyses. AD, axial diffusivity; FA, fractional anisotropy; MD, mean diffusivity; MTR, magnetization transfer ratio; RD, radial diffusivity

After multivariable analyses, MD and AD were found to be significantly different between neurological grades (Figure 4). Mean diffusivity was significantly lower in grade 5 compared to grades 2 (*P* = .02) and 3 (*P* = .04), and in grade 4 compared to grade 2 (*P* = .03). Axial diffusivity was significantly lower in grade 5 compared to grades 2 (*P* < .001) and 3 (*P* < .001), and in grade 4 compared to grade 3 (*P* = .003).

## 4 DISCUSSION

Our study prospectively evaluated the use of QMRI in dogs with thoracolumbar IVDH. Mean diffusivity, AD, and RD had a positive correlation with age and weight. Fractional anisotropy had a negative correlation with age. We also observed that MTR, FA, MD, and RD values in dogs with acute SCI were significantly different from those with chronic SCI caused by IVDH, and that MTR and MD were significantly associated with the affected spinal cord segment. Fractional anisotropy was found to be significantly lower in dogs where a T2W‐IH was present compared to dogs without at T2W‐IH. On multivariable analyses, MD and RD were significantly correlated with neurological grade, being lower in dogs with grade 5 neurologic dysfunction compared to grades 2 and 3.

Diffusion tensor imaging utilizes MRI to noninvasively measure the molecular diffusion of water in vivo using bipolar magnetic field gradient pulses.^26^ As water molecules are able to diffuse 3‐dimensionally within a body system, DTI is able to detect not only the magnitude but also the anisotropy of water molecules in the x, y, and z directions.^26^ Diffusion tensor imaging therefore provides information about the microstructure and physiologic state of body systems, and changes in the magnitude or anisotropy of water molecules are the result of obstacles limiting the movement of water molecules (change in magnitude), or disarrangement of the body system (change of anisotropy).^26^, ^27^ Indices of DTI are difficult to display as images, therefore DTI measurements of water molecules are commonly calculated by diffusion ellipsoids (*λ*) which represent the average 3‐dimensional diffusion over a given distance over time^28^ where *λ*_1_ characterizes x, *λ*_2_ characterizes y, and *λ*_3_ characterizes z.^26^

Fractional anisotropy represents scalar values, from 0 to 1, and provides directionality information of the anisotropic diffusion of water molecules at each voxel.^29^ An FA value of 0 would represent complete isotropy or unrestricted diffusion of water. Within the CNS, this would be expected in areas such as the ventricles.^17^ An FA value closer to 1 would represent anisotropy where diffusion is greater in 1 direction, *λ*_1_, compared to the other directions, *λ*_2_ and *λ*_3_.^17^ In our study, the quantitative DTI parameter FA was not significantly different in dogs with more severe neurologic dysfunction. Interestingly, a recent study on SCI in dogs found FA to be higher in dogs with a worse outcome.^30^ For our study, we did find a trend for FA to increase in dogs with a higher neurologic grade when a T2W‐IH was absent, and the opposite when a T2W‐IH was present. Comparing our findings to Wang‐Leandro et al, a possible explanation for FA being higher in dogs with a worse outcome could be related to the effects of T2W‐IH on FA.^30^ In our study, FA was significantly lower in dogs with a T2W‐IH when compared to dogs without at T2W‐IH. It has been shown that a T2W‐IH is associated with a worse outcome in dogs with IVDH.^6^ Fractional anisotropy has been shown to change if a T2W‐IH is present.^31^ In rats, FA will increase with vasogenic edema then return to normal values once the edema resolves.^17^ Therefore, the FA being decreased in our population of dogs could be related to other causes of T2W‐IH other than edema including: gliosis, inflammation, hemorrhage, or malacia. Fractional anisotropy was also found to be lower in dogs with chronic SCI compared to acute SCI. This correlates to a previous study evaluating experimental SCI in dogs.^32^ Axonal injury is known to decrease FA values.^33^ A possible explanation for the difference in FA values between chronic and acute SCI is the effects of vasogenic edema on FA compared to the effects of chronic axonal injury. However, more information on FA is needed to confirm these conclusions.

Directional magnitude of water molecules can be measured by MD, AD, and RD. The most extensively studied metric of directional magnitude in dogs is the apparent diffusion coefficient, for which DTI is similar to MD, although AD and RD have also been evaluated in dogs with SCI.^33^, ^34^, ^35^, ^36^ A significant increase in MD has been correlated to severe axonal degeneration in dogs with SCI^34^ thought to be associated with demyelination, inflammation, and edema.^37^ A decrease in MD represents an increase in resistance to diffusion rates.^37^ In our study, the DTI measurements MD and AD were significantly lower in dogs with more severe neurologic dysfunction after accounting for the effects that age, body weight, duration of SCI, and longitudinal location of IVDH were demonstrated to have on these metrics.

Studies on SCI suggest that acute injury compared to chronic injury may exhibit different cellular response which can alter DTI measurements.^15^ With severe acute injury, MD and RD will generally decrease and with chronic injury it will tend to increase.^15^ Axial diffusivity should not to be affected by the chronicity of disease.^34^ In our population of dogs, MD and RD were significantly lower in dogs with acute compared to dogs with chronic IVDH consistent with what has been previously published.^15^ Axial diffusivity was not significantly different in dogs with acute compared to dogs with chronic IVDH. Chronic SCI is generally associated with demyelination and axonal loss, along with gliosis and syrinx formation.^15^ Therefore, with chronic SCI diffusion will increase but anisotropy will decrease, leading to the increase in MD and RD seen with chronic IVDH, and AD being unaffected.^15^ Acute SCI is often associated with ischemia, contusion, and inflammation leading to lower diffusion values.^15^

A positive correlation was found with age, body weight, and MD, AD and RD. In people, MD, AD, and RD have been shown to increase with age and body mass index, which suggests there is a decline in white matter composition and integrity related to age and weight.^38^, ^39^ This leads to a decrease in anisotropy and increase in isotropy as there is loss in the white matter volume. In this population of dogs, a negative correlation was seen between FA and age which is also present in human patients likely associated to a decline in white matter composition and integrity similar to MD, AD, and RD.^38^

Our study also prospectively evaluated the use of MTR in dogs with IVDH. Magnetization transfer ratio was not significantly different between neurologic grades but was found to be significantly lower in dogs with chronic compared to acute injury. To the author's knowledge, this is the first study to evaluate MTR in dogs with SCI. Magnetization transfer ratio measures the ability of low protein fluid to exchange magnetization, and alterations to MTR correlate to myelination state.^9^, ^12^ Values of MTR vary depending on the time frame of imaging with MTR increasing within the first 2 weeks of CNS injury, and between 16 and 28 days this will reverse to a reduction in MTR.^16^ Although the reason for the rise in MTR is not well understood, it is likely related to the stages of Wallerian degeneration and acute secondary injury, and is not related to edema.^16^ Instead, there is an increase in available proton exchange sites which can occur with axonal membranes collapsing into ellipsoid bodies and a physiochemical change in the myelin lipid bilayer altering the availability of cholesterol for relaxation exchange.^16^

Our study did not find a significant difference in MTR when compared to severity of neurologic dysfunction. Comparing MTR with acute, subacute, and chronic SCI, we found MTR to be significantly lower in dogs with chronic SCI compared to dogs with acute SCI. Experimental data suggest that chronic CNS lesions might have a lower MTR, similar to our study, thought to be related to myelin loss.^16^ Although MTR does not appear helpful in differentiating neurologic grades in dogs with thoracolumbar IVDH, it could be helpful in differentiating acute from chronic SCI and warrants further investigation.

In our study, we elected to use SCT for MTR and DTI measurements. Spinal Cord Toolbox is able to automatically calculate MTR, FA, MD, AD, and RD, allowing for better consistency in processing MTR and DTI measurements in comparison to ROI.^22^ It is an open source software which utilizes an atlas‐based system to properly capture the spinal cord shape, including gray matter and white matter.^40^ Spinal Cord Toolbox has been utilized in cats and therefore shows promise in dogs.^41^ We were able to analyze all QMRI measurements on each dog with SCT. Motion correction methods for DTI are utilized by the SCT software. All images can be evaluated with FSLview after processing to inspect that the software is working correctly.

One limitation of the study is that MTR was not performed on any normal dogs, and therefore we cannot make a conclusion of how important the change of MTR was compared to normal dogs. It was also found that MTR and DTI measurements varied by body weight, age and spinal cord segment. The significance of this is unknown but stresses the importance of age and body weight matched controls in future studies, along with normal values for each spinal cord segment.

In our study, a *b*‐value of 800 was used for DTI. Although we were able to perform quantitative DTI measurements on all dogs, only 1 dog had a cranial thoracic lesion. We were still able to perform DTI on this dog without the use of gating; however, we cannot make a conclusion of how practical this would be on dogs with mid thoracic lesions. Comparing *b*‐values of 500 and 800 could be helpful in the future as a *b*‐value of 500 would likely lead to less artifact and might be more reliable in the thoracic spinal cord region.

This is also the first study to use SCT in dogs. The atlas used for the analysis in this population of dogs was based off of the human spine and spinal cord. Variations do exist between the gray and white matter of the species. Ideally, an atlas should be made for SCT using the dog.

## 5 CONCLUSION

Mean diffusivity and AD were significantly different between neurological grades, and could serve as surrogates of SCI severity. These measurements are complementary to the clinical exam in dogs with thoracolumbar IVDH. Additional studies with larger samples sizes are needed to further assess how QMRI techniques could be helpful in determining long‐term prognosis in dogs with IVDH, taking into account how age, the temporal duration of SCI, body weight, spinal cord segment, and T2W‐IH affect QMRI measurements.

## ACKNOWLEDGMENTS

We thank Julien Cohen‐Adad, PhD Associate Professor of Biomedical Engineering, Polytechnique Montreal for his help with Spinal Cord Toolbox.

## CONFLICT OF INTEREST DECLARATION

Authors declare no conflict of interest.

## OFF‐LABEL ANTIMICROBIAL DECLARATION

Authors declare no off‐label use of antimicrobials.

## INSTITUTIONAL ANIMAL CARE AND USE COMMITTEE (IACUC) OR OTHER APPROVAL DECLARATION

This study was approved by the Department of Small Animal Clinical Sciences at the Virginia‐Maryland College of Veterinary Medicine.

## HUMAN ETHICS APPROVAL DECLARATION

Authors declare human ethics approval was not needed for this study.

## References

[1] Bergknut N , Egenvall A , Hagman R , et al. Incidence of intervertebral disk degeneration‐related diseases and associated mortality rates in dogs. J Am Vet Med Assoc. 2012;240(11):1300‐1309. 22607596 10.2460/javma.240.11.1300

[2] Henke D , Vandevelde M , Doherr MG , Stöckli M , Forterre F . Correlations between severity of clinical signs and histopathological changes in 60 dogs with spinal cord injury associated with acute thoracolumbar intervertebral disc disease. Vet J. 2013;198(1):70‐75. 23702280 10.1016/j.tvjl.2013.04.003

[3] Olby NJ , Lim J , Wagner N , et al. Time course and prognostic value of serum GFAP, pNFH, and S100β concentrations in dogs with complete spinal cord injury because of intervertebral disc extrusion. J Vet Intern Med. 2019;33(2):726‐734. 30758078 10.1111/jvim.15439 PMC6430936

[4] Leigh EJ , Mackillop E , Robertson ID , Hudson LC . Clinical anatomy of the canine brain using magnetic resonance imaging. Vet Radiol Ultrasound. 2008;49(2):113‐121. 18418990 10.1111/j.1740-8261.2008.00336.x

[5] Levine JM , Fosgate GT , Chen AV , et al. Magnetic resonance imaging in dogs with neurologic impairment due to acute thoracic and lumbar intervertebral disk herniation. J Vet Intern Med. 2009;23(6):1220‐1226. 19780928 10.1111/j.1939-1676.2009.0393.x

[6] Ito D , Matsunaga S , Jeffery ND , et al. Prognostic value of magnetic resonance imaging in dogs with paraplegia caused by thoracolumbar intervertebral disk extrusion: 77 cases (2000‐2003). J Am Vet Med Assoc. 2005;227(9):1454‐1460. 16279391 10.2460/javma.2005.227.1454

[7] Penning V , Platt SR , Dennis R , Cappello R , Adams V . Association of spinal cord compression seen on magnetic resonance imaging with clinical outcome in 67 dogs with thoracolumbar intervertebral disc extrusion. J Small Anim Pract. 2006;47(11):644‐650. 17076787 10.1111/j.1748-5827.2006.00252.x

[8] Jeffery ND , Barker AK , Hu HZ , et al. Factors associated with recovery from paraplegia in dogs with loss of pain perception in the pelvic limbs following intervertebral disk herniation. J Am Vet Med Assoc. 2016;248(4):386‐394. 26829270 10.2460/javma.248.4.386

[9] McGowan JC , Berman JI , Ford JC , Lavi E , Hackney DB . Characterization of experimental spinal cord injury with magnetization transfer ratio histograms. J Magn Reson Imaging. 2000;12(2):247‐254. 10931587 10.1002/1522-2586(200008)12:2<247::aid-jmri6>3.0.co;2-x

[10] Kim JH , Loy DN , Wang Q , et al. Diffusion tensor imaging at 3 hours after traumatic spinal cord injury predicts long‐term locomotor recovery. J Neurotrauma. 2010;27(3):587‐598. 20001686 10.1089/neu.2009.1063 PMC2867549

[11] Wolff SD , Balaban RS . Magnetization transfer contrast (MTC) and tissue water proton relaxation in vivo. Magn Reson Med. 1989;10(1):135‐144. 2547135 10.1002/mrm.1910100113

[12] Filippi M , Rocca MA . Magnetization transfer magnetic resonance imaging of the brain, spinal cord, and optic nerve. Neurotherapeutics. 2007;4(3):401‐413. 17599705 10.1016/j.nurt.2007.03.002 PMC7479733

[13] Cloney MB , Smith ZA , Weber KA , Parrish TB . Quantitative magnetization transfer MRI measurements of the anterior spinal cord region are associated with clinical outcomes in cervical spondylotic myelopathy. Spine. 2018;43(10):675‐680. 29068880 10.1097/BRS.0000000000002470 PMC6621550

[14] Winklewski PJ , Sabisz A , Naumczyk P , Jodzio K , Szurowska E , Szarmach A . Understanding the physiopathology behind axial and radial diffusivity changes‐what do we know? Front Neurol. 2018;9:92. 29535676 10.3389/fneur.2018.00092 PMC5835085

[15] Yoon H , Moon W‐J , Nahm S‐S , Kim J , Eom K . Diffusion tensor imaging of scarring, necrosis, and cavitation based on histopathological findings in dogs with chronic spinal cord injury: evaluation of multiple diffusion parameters and their correlations with histopathological findings. J Neurotrauma. 2018;35(12):1387‐1397. 29298564 10.1089/neu.2017.5409

[16] Lexa FJ , Grossman RI , Rosenquist AC . Dyke award paper. MR of wallerian degeneration in the feline visual system: characterization by magnetization transfer rate with histopathologic correlation. AJNR Am J Neuroradiol. 1994;15(2):201‐212. 8192062 PMC8334622

[17] Kimura‐Ohba S , Yang Y , Thompson J , et al. Transient increase of fractional anisotropy in reversible vasogenic edema. J Cereb Blood Flow Metab. 2016;36(10):1731‐1743. 26865662 10.1177/0271678X16630556 PMC5076788

[18] Levine JM , Ruaux CG , Bergman RL , Coates JR , Steiner JM , Williams DA . Matrix metalloproteinase‐9 activity in the cerebrospinal fluid and serum of dogs with acute spinal cord trauma from intervertebral disk disease. Am J Vet Res. 2006;67(2):283‐287. 16454634 10.2460/ajvr.67.2.283

[19] McKee M . Intervertebral disc disease in the dog 1. Pathophysiology and diagnosis. In Pract. 2000;22(7):355‐369.

[20] De Risio L , Muñana K , Murray M , Olby N , Sharp NJH , Cuddon P . Dorsal laminectomy for caudal cervical spondylomyelopathy: postoperative recovery and long‐term follow‐up in 20 dogs. Vet Surg. 2002;31(5):418‐427. 12209412 10.1053/jvet.2002.34673

[21] Levine GJ , Levine JM , Budke CM , et al. Description and repeatability of a newly developed spinal cord injury scale for dogs. Prev Vet Med. 2009;89(1‐2):121‐127. 19303151 10.1016/j.prevetmed.2009.02.016

[22] De Leener B , Lévy S , Dupont SM , et al. SCT: spinal cord toolbox, an open‐source software for processing spinal cord MRI data. NeuroImage. 2017;145(pt A):24‐43. 27720818 10.1016/j.neuroimage.2016.10.009

[23] Miyanji F , Furlan JC , Aarabi B , Arnold PM , Fehlings MG . Acute cervical traumatic spinal cord injury: MR imaging findings correlated with neurologic outcome—prospective study with 100 consecutive patients. Radiology. 2007;243(3):820‐827. 17431129 10.1148/radiol.2433060583

[24] Moore SA , Granger N , Olby NJ , et al. Targeting translational successes through CANSORT‐SCI: using pet dogs to identify effective treatments for spinal cord injury. J Neurotrauma. 2017;34(12):2007‐2018. 28230415 10.1089/neu.2016.4745 PMC5467140

[25] Saksena S , Middleton DM , Krisa L , et al. Diffusion tensor imaging of the normal cervical and thoracic pediatric spinal cord. Am J Neuroradiol. 2016;37(11):2150‐2157. 27418470 10.3174/ajnr.A4883 PMC7963763

[26] Le Bihan D , Mangin JF , Poupon C , et al. Diffusion tensor imaging: concepts and applications. J Magn Reson Imaging. 2001;13(4):534‐546. 11276097 10.1002/jmri.1076

[27] Basser PJ , Mattiello J , LeBihan D . Estimation of the effective self‐diffusion tensor from the NMR spin echo. J Magn Reson B. 1994;103(3):247‐254. 8019776 10.1006/jmrb.1994.1037

[28] Basser PJ , Mattiello J , LeBihan D . MR diffusion tensor spectroscopy and imaging. Biophys J. 1994;66(1):259‐267. 8130344 10.1016/S0006-3495(94)80775-1 PMC1275686

[29] Hobert MK , Stein VM , Dziallas P , Ludwig DC , Tipold A . Evaluation of normal appearing spinal cord by diffusion tensor imaging, fiber tracking, fractional anisotropy, and apparent diffusion coefficient measurement in 13 dogs. Acta Vet Scand. 2013;55:36. 23618404 10.1186/1751-0147-55-36 PMC3648354

[30] Wang‐Leandro A , Siedenburg JS , Hobert MK , et al. Comparison of preoperative quantitative magnetic resonance imaging and clinical assessment of deep pain perception as prognostic tools for early recovery of motor function in paraplegic dogs with intervertebral disk herniations. J Vet Intern Med. 2017;31(3):842‐848. 28440586 10.1111/jvim.14715 PMC5435037

[31] Svärd D , Nilsson M , Lampinen B , et al. The effect of white matter hyperintensities on statistical analysis of diffusion tensor imaging in cognitively healthy elderly and prodromal Alzheimer's disease. PLoS One. 2017;12(9):e0185239. 28934374 10.1371/journal.pone.0185239 PMC5608410

[32] Liu C‐B , Yang D‐G , Meng Q‐R , et al. Dynamic correlation of diffusion tensor imaging and neurological function scores in beagles with spinal cord injury. Neural Regen Res. 2018;13(5):877‐886. 29863019 10.4103/1673-5374.232485 PMC5998642

[33] Lin M , He H , Schifitto G , Zhong J . Simulation of changes in diffusion related to different pathologies at cellular level after traumatic brain injury. Magn Reson Med. 2016;76(1):290‐300. 26256558 10.1002/mrm.25816 PMC4769952

[34] Yoon H , Kim J , Moon W‐J , et al. Characterization of chronic axonal degeneration using diffusion tensor imaging in canine spinal cord injury: a quantitative analysis of diffusion tensor imaging parameters according to histopathological differences. J Neurotrauma. 2017;34(21):3041‐3050. 28173745 10.1089/neu.2016.4886

[35] Konishi Y , Satoh H , Kuroiwa Y , et al. Application of fiber tractography and diffusion tensor imaging to evaluate spinal cord diseases in dogs. J Vet Med Sci. 2017;79(2):418‐424. 28025450 10.1292/jvms.16-0504 PMC5326951

[36] Lewis MJ , Yap P‐T , McCullough S , Olby NJ . The relationship between lesion severity characterized by diffusion tensor imaging and motor function in chronic canine spinal cord injury. J Neurotrauma. 2018;35(3):500‐507. 28974151 10.1089/neu.2017.5255 PMC5793950

[37] Shanmuganathan K , Zhuo J , Chen HH , et al. Diffusion tensor imaging parameter obtained during acute blunt cervical spinal cord injury in predicting long‐term outcome. J Neurotrauma. 2017;34(21):2964‐2971. 28385062 10.1089/neu.2016.4901

[38] Rathee R , Rallabandi VPS , Roy PK . Age‐related differences in white matter integrity in healthy human brain: evidence from structural MRI and diffusion tensor imaging. Magn Reson Insights. 2016;9:9‐20. 27279747 10.4137/MRI.S39666 PMC4898444

[39] Verstynen TD , Weinstein AM , Schneider WW , Jakicic JM , Rofey DL , Erickson KI . Increased body mass index is associated with a global and distributed decrease in white matter microstructural integrity. Psychosom Med. 2012;74(7):682‐690. 22879428 10.1097/PSY.0b013e318261909c PMC3586991

[40] Prados F , Ashburner J , Blaiotta C , et al. Spinal cord grey matter segmentation challenge. NeuroImage. 2017;152:312‐329. 28286318 10.1016/j.neuroimage.2017.03.010 PMC5440179

[41] De Leener B , Mangeat G , Dupont S , et al. Topologically preserving straightening of spinal cord MRI. J Magn Reson Imaging. 2017;46(4):1209‐1219. 28130805 10.1002/jmri.25622

